# Aphasia Rehabilitation in India: Current Practices and Future Directions

**DOI:** 10.3390/healthcare14040434

**Published:** 2026-02-09

**Authors:** Sunil Kumar Ravi, Sai Samyuktha Vachavai, Saraswathi Thupakula, Irfana Madathodiyil, Vijaya Kumar Narne, Krishna Yerraguntla, Abdulaziz Almudhi, Deepak Puttanna, Abhishek Budiguppe Panchakshari

**Affiliations:** 1 Department of Medical Rehabilitation Sciences, College of Applied Medical Sciences, King Khalid University, Abha 61481, Saudi Arabia; imadathodiyil@kku.edu.sa (I.M.); vnarne@kku.edu.sa (V.K.N.); ksuryanarayana@kku.edu.sa (K.Y.); almudhi@kku.edu.sa (A.A.); 2 Speech-Language Pathology Unit, College of Applied Medical Sciences, King Khalid University, Abha 61481, Saudi Arabia; 3 Department of Speech Language Pathology, Shravana Institute of Speech and Hearing, Vijayanagara Sri Krishnadevaraya University, Ballari 583102, India; samyukthav408@gmail.com (S.S.V.); saraswathi.aslp@gmail.com (S.T.); 4 Department of Audiology and Speech Language Pathology, Taibah University, Madinah 42353, Saudi Arabia; drangaiah@taibahu.edu.sa; 5 Center of Speech Language Sciences, All India Institute of Speech and Hearing, Mysore 570006, India; abhishekbp@aiishmysore.in

**Keywords:** aphasia rehabilitation, India, speech-language pathologists, bilingualism, clinical challenges, tele-rehabilitation, assessment tools, patient education

## Abstract

**Highlights:**

**What are the main findings?**

- The present study results revealed significant challenges in formal assessment of aphasia due to cultural and linguistic barriers, time constraints, and patient cooperation issues.
- The results also revealed limited clinician’s perceived knowledge, confidence, and use of specific approaches other than localization approach. Further, the results also indicated significant challenges while treating bilingual and multilingual patients.
- On the factors related to patients, clinicians reported limited resources, higher expectations, patient motivation as major factors in aphasia rehabilitation.

**What are the implications of the main findings?**

- Indian aphasiology research studies may immediately address the need for developing reliable and valid formal screening and diagnostic tools that are culturally and linguistically appropriate.
- There is a greater need for educating and clinical training on the recent approaches to the clinicians to enable them to use wide range of techniques.

**Abstract:**

**Background/Objectives:** The Speech-Language Pathologists (SLP) are an integral part of the multidisciplinary team approach to rehabilitation of persons with aphasia (PWA). However, the efficacy of treatment provided by SLPs can vary due to several factors related to clinicians, patients, and the availability of services. The present study was conducted with the aim of investigating current practices in aphasia rehabilitation, key challenges, and future directions as perceived by the SLPs in the Indian context. **Methods:** The study was conducted using a web-based survey comprising a 32-item questionnaire to gather information related to demographic and professional details, knowledge and use of aphasia rehabilitation approaches, patient education, counselling, bilingual & multilingual contexts, and challenges faced by SLPs. A total of 142 responses were analyzed after initial screening to assess the knowledge, use, and confidence of aphasia rehabilitation along with challenges faced by SLPs in the Indian context. **Results:** The results indicated significant challenges in the assessment of aphasia due to a lack of formal screening and diagnostic languages in several languages. Further, the results also indicated variations in the knowledge level and confidence in the use of various approaches to aphasia rehabilitation, which warrants the urgent need for organizing short-term training programs for SLPs. The participants also self-reported significant challenges in managing bilingual and multilingual patients with aphasia due to differences in their knowledge and confidence in the selection of language for treatment. On the other side, major patient-related challenges include inadequate logistics, lack of funding, unavailability of speech and language therapy services, social acceptance, and support from family members. The participants also reported the necessity of improving tele-rehabilitation services and developing materials and mobile apps for rehabilitation in Indian languages as future directions for aphasia rehabilitation. **Conclusions:** The present study through a self-reported questionnaire identified key challenges in aphasia rehabilitation related to the clinician and PWA in the Indian context. The results of the study warrant the need for immediate action to overcome the challenges to enhance the rehabilitation services to PWAs.

## 1. Introduction

Aphasia is an acquired neurogenic language disorder that impairs various modalities of language such as expressive language, receptive language, reading and writing due to acquired brain injury to the dominant hemisphere. Aphasia is one of the frequently seen disorders in stroke survivors associated with hemiplegia, sensory disturbances, etc. Research studies in the past estimated that approximately one million individuals in the United States, around 80,000 in Australia, and 250,000 in Britain are affected with post-stroke aphasia [1,2,3]. The incidence and prevalence of aphasia in India appear to be significantly higher than in Western contexts, with studies indicating that 21–38% of stroke survivors in India are affected by aphasia, amounting to an estimated two million individuals [4]. Cerebrovascular disease or stroke is reported to be the primary cause of aphasia; however, it may also result from traumatic brain injury, tumors, and other neuropathological conditions. Persons with aphasia (PWA) may encounter substantial challenges in various language domains, including expressive language, receptive language, naming, repetition, reading, and writing, contingent upon the degree and location of the lesion. Research on spontaneous recovery in aphasia indicates that the most significant recovery occurs within the initial three months post-onset even in severe aphasia, followed by subtle improvement up to nine months, ultimately culminating in a plateau [5,6,7], although depends on several predictive factors such as initial severity [5], site of lesion [8,9], lesion size [4,10], age [11,12,13], among others. Nonetheless, the majority of PWA encounter challenges with at least one language component throughout their lives, which may negatively impact both their quality of life and that of their caretakers [7,14,15,16,17].

The vast majority of research studies on aphasia rehabilitation have indicated a significant impact of speech and language therapy at acute stages on persons’ recovery from aphasia, consequently enhancing the quality of life for individuals with aphasia [18,19,20,21,22,23]. However, several variables hinder the recovery of PWA, including treatment intensity, duration, patient motivation, and the availability of resources and services [24,25].

Several studies carried out worldwide in the past have highlighted the various challenges in aphasia rehabilitation services and they are classified into clinician-related, and patient-related issues. A multinational comparison study conducted by Katz et al. [26] examined aphasia rehabilitation practices across five healthcare systems: the Private Sector in the United States, Veteran Affairs in the United States, the United Kingdom, Australia, and Canada. The findings revealed that 49% of centers in the United States offer group treatment, while Australia reported the lowest percentage at 24% of centers. Conversely, a notably greater number of sessions were offered to acute patients in Canada, the United States–Private Sector, and the United States–Veteran Affairs, in contrast to the UK and Australia, which provided only one to five sessions at the acute stage. Upon the conclusion of treatment, most respondents indicated that the cessation was attributed to the “patient achieving all goals of treatment”. In contrast, a minority cited “no progress” and “treatment no longer reimbursed” as reasons for the termination of treatment across various countries. A query regarding follow-up practices indicated that 53% of clinicians engage in follow-up with their patients after discharge either ‘some of the time’ or ‘never.’

Similarly, a study conducted by Rose et al. [2] examined the practices of aphasia rehabilitation in Australia, highlighting considerable challenges faced by clinicians when patients experience dysphagia and physical impairments as related issues in acute care. Conversely, clinicians indicated that group therapy services are underutilized in their practice, primarily due to funding challenges, which are identified as a significant obstacle to the advancement of intensive aphasia rehabilitation services. Notable obstacles were recognized in the realm of aphasia rehabilitation within community settings, particularly stemming from issues like a shortage of professionals, transportation difficulties, and spatial limitations in rural regions. While the study participants indicated a strong understanding, confidence, and application of diverse approaches for aphasia rehabilitation, they also highlighted a critical shortage of sufficient resources, overwhelming workloads, and elevated stress levels as major personal challenges.

The Indian context of aphasia rehabilitation poses various challenges due to large population, limited awareness among PWAs, inadequate number of rehabilitation centers for aphasia rehabilitation, financial burden, and many others. The demographics of India reveal that a significant portion of country’s population lives in rural and semi-urban areas with limited awareness about the treatment services for PWA [27]. Further, factors such as improper referrals, societal attitudes, accessibility of speech and language therapy services, and economic considerations significantly influence the rehabilitation of PWA in Indian context. Consequently, these factors lead to elevated discontinuation rates from rehabilitation services nationwide [4].

One of the significant challenges in aphasia rehabilitation across globe and also India is the availability of SLPs and their knowledge, skill levels in offering therapy to PWA. The rehabilitation for PWA involves a multidisciplinary team approach with Speech-Language Pathologists playing an important role. Speech-language therapy services commenced in the early 1960s in India with the establishment of distinguished institutions providing graduate and post-graduate programs in speech-language pathology. Presently, over 6000 audiologists and speech therapists are registered with the Rehabilitation Council of India, a statutory body under the Ministry of Social Justice and Empowerment, Government of India, which regulates training programs at both the bachelor’s and master’s levels in speech-language pathology and is responsible for licensing qualified graduates to practice as audiologists and speech therapists in India.

According to the Rehabilitation Council of India Act, 1992, a Bachelor’s degree in Speech Language Pathology and Audiology is the primary qualification necessary to practice as an independent speech therapist in India, although many graduates pursue post-graduate studies in Speech Language Pathology. The disparities in educational qualifications and the quality of academic and clinical training at various universities in India may significantly affect the overall quality of aphasia rehabilitation services they offer, owing to inequalities in their knowledge, skill level, confidence for various treatment approaches.

Several challenges exist for speech–language pathologists in the rehabilitation of aphasia within the Indian environment as reported by previous studies. Firstly, there is a lack of standardized assessment instruments for the thorough evaluation of aphasia at both acute and chronic stages, despite the translation and adaptation of a limited number of tests into Indian languages, such as the Western Aphasia Battery–Revised [28]. Nonetheless, the psychometric features of these translated test batteries remain inadequately validated in numerous languages. India’s linguistic diversity encompasses 22 official languages and over 1600 dialectal variants, presenting substantial challenges for speech–language pathologists in doing complete assessments for PWA [4,29,30,31]. In addition, several obstacles encompass insufficient time for clinicians resulting from excessive demand, inadequate facilities, patients’ desire, and a deficiency in clinical training among graduates, all of which significantly impede aphasia rehabilitation in the Indian environment [4,32].

Very few studies have been conducted in Indian context to understand the practices and challenges in aphasia rehabilitation. In a study by Tiwari and Krishnan [32] conducted a survey study on aphasia rehabilitation in India, highlighting significant client-related concerns such as poor economic status, distant therapy services, inadequate family support, lack of motivation, associated conditions, and a general lack of awareness. The authors identified several factors, including time constraints and the overall inefficiency of therapy techniques, as significant clinician-related concerns within the Indian context. Additionally, the study findings indicated that the absence of epidemiological data, challenges related to multilingualism, illiteracy, limited access to rehabilitation centers, and the unavailability of aphasia support groups are significant obstacles to aphasia rehabilitation in the Indian context. Although the results of this study reported major challenges in Indian context, there are no recent studies to understand the current practices and challenges.

The Expert Group Meeting on Aphasia [4] highlighted the importance and need for standardized, culturally and linguistically appropriate assessment tools for assessment of aphasia. Further, the group also recommended the need for developing standardized protocols and treatment manuals specifically in Indian languages. Additionally, the Expert group also provided several action plans such as conducting epidemiological surveys on prevalence of aphasia; plan and conduct training workshops to healthcare professionals involved in aphasia rehabilitation such as SLPs, neurologists, clinical psychologists, and others; encourage designing and development of digital assessment batteries, development of mobile applications and software programs to implement tele-therapy services in remote areas.

Considering these complex issues, there is an urgent necessity for research that investigates the current practices, challenges, and explores future directions for aphasia rehabilitation in Indian context as perceived by the SLPs. Understanding these significant challenges in both clinician-oriented and client-oriented domains is essential for devising future aphasia rehabilitation strategies in any nation, including the Indian context. The review of literature distinctly highlights a paucity of research confronting these challenges within Indian context, except for a study conducted by Tiwari and Krishnan [32]. Consequently, the present study was carried out as an exploratory survey to examine the perceived levels of clinical competence with respect to knowledge, confidence and use of treatment approaches, current practices, and perceived challenges along with future directions for aphasia rehabilitation in Indian context as perceived by Speech language Pathologists (SLPs).

## 2. Materials and Methods

### 2.1. Questionnaire Development and Validation

A 45–item questionnaire was initially developed by the authors based on insights from the study by Rose et al. [2] under six domains namely, (1) Demographic details; (2) knowledge and use of approaches to aphasia rehabilitation; (3) importance of patient education, counselling and follow-up practices; (4) Communication access, availability of aphasia support groups; (5) Bilingual and multilingualism; (6) Challenges in aphasia rehabilitation practices. As part of validation process of the questionnaire, the questionnaire was given to five speech language pathologists with more than 10 years of experience in working with PWA in different parts of India. Based on the feedback from the validation process and based on suggestions from the SLPs, the final questionnaire was prepared with 32 items under five sections:

- Demographic and professional details (10 items)
- Knowledge and use of aphasia rehabilitation approaches (11 items)
- Importance of patient education, counseling, and follow-up (2 items)
- Bilingual and multilingual contexts (3 items)
- Challenges to aphasia rehabilitation in the Indian context (6 items)

The final questionnaire was sent to five independent SLPs with PhD in the field with experience of working with PWAs to review the questionnaire for content validity. For content validity, Survey Instrument Validation Questionnaire [33] was used which consists of 14 items with each item rated between 1 (strongly disagree) to 5 (strongly agree) for the overall questionnaire. The content validity index for the questionnaire (S-CVI) was calculated by averaging the cumulative level of agreement between the experts. The analysis revealed S-CVI Scores ranging from 0.85 to 1.0 for the questionnaire.

The questionnaire comprised of various forms of items such as yes/no questions, ratings scales, and open-ended questions. The questionnaire consists of few items related to the knowledge (knowing or describing about an approach), confidence (self-efficiency in delivering an approach), and use (actual use of an approach in clinical settings) of various approaches in aphasia rehabilitation on a 5-point Likert scale. As the objectives of the present study were to explore the current practices, challenges and future directions, additional importance was given to these factors in the questionnaire. The questionnaire was uploaded on to Google Forms and was distributed electronically to approximately 1800 speech therapists who have valid registration with Rehabilitation Council of India between September 2024 to November 2024 through emails, social media platforms, and through institutions across India. The ethical clearance was obtained from the IEC of King Khalid University (ECM #2024-3107) and from the IEC of Shravana Institute of Speech and Hearing, India for the study.

### 2.2. Participants

A total of 198 responses were received from speech therapists within the given time period. Of the 197 speech therapists who participated in survey, 53 speech therapists reported that they are working with other speech and language disorders such as child language disorders, fluency disorders, dysphagia, etc and these responses were removed from the final analysis. Another 2 participants did not provide consent for participation in the study and hence they were removed from the final analysis. Following an initial screening, a total of 142 responses were included for the final analysis of the study. All the participants of the study were given a gift voucher from Amazon India as compensation for their time only after completion of the survey to avoid any potential effects on the results.

The analysis of demographic data revealed that the sample included 32.4% male (n = 46) and 67.6% female (n = 96) speech therapists with mean age of 30.28 years and 27.52 years for male and female groups respectively. The mean experience of participants was 6.13 years and 4.30 years for male and female groups respectively. Further, 43.7% of participants had Bachelors’ degree in Speech-language Pathology (n = 62), 52.1% had master’s degree in speech-language pathology, and 4.2% of participants had Doctoral degree in Speech-language pathology. Of the total participants, 53.5% of participants were from cities (n = 76), 32.4% of participants from metro cities (n = 46), 12.0% of participants from towns (n = 17), and remaining 2.1% of participants were from rural areas (n = 3) indicating wide range distribution of sample. Further, 33.8% of participants worked in multiple settings (n = 48), 20.4% of participants worked in hospital setting (n = 29), 21.9% of participants worked in independent speech and language clinic (n = 31), 14.1% of participants worked as clinician in academic institutions (n = 20), 7.7% of participants worked as teaching faculty in academic institutions, and 2.1% of participants have worked as researchers in aphasia (n = 3). For question on average number of patients with aphasia seen by SLPs, 62.7% of participants reported that they see 0 to 5 aphasia patients; 21.1% reported 6 to 10 aphasia patients; 7% of participants reported 11 to 15 aphasia patients; and 9.2% of participants reported above 15 aphasia patients on an average in a month.

### 2.3. Data Analysis

The response data of participants were downloaded from google forms and collated into Excel database for further data processing. Data was screened on the basis of consent for the study, type of clinical case load and excluded the data of participants who did not serve aphasia patients in their clinical practice. Data was also screened for any missing data, duplication, and the validity of responses for open-ended questions for each participant. The data was coded with numerical values for questions on knowledge (5—very good; 4—good; 3—adequate; 2—limited; 1—very limited), confidence (5—very confident; 4—confident; 3—neutral; 2—unconfident; 1—very unconfident), use (5—very frequently; 4—frequently; 3—occasionally; 2—rarely; 1—very rarely) for the purpose of descriptive statistics. This categorization facilitated a systematic examination of patterns of therapists’ knowledge, confidence, and practice systems with regard to aphasia rehabilitation. The responses of the participants to the open-ended questions were qualitatively analyzed collaboratively by two independent SLPs with more than 10 years of clinical experience in the field of aphasiology and were not involved in the study using content analysis method. The responses which were agreed by both reviewers using content analysis were reported in the results, while other responses were discarded. Multivariate General Linear Model (GLM) was performed using R Statistical Software (v4.3.3; 2024) for examining predictors of constructs (Knowledge, confidence and use levels) with gender, workplace setting, academic qualifications, experience.

## 3. Results

### 3.1. Knowledge and Use of Approaches to Aphasia Rehabilitation

A total of 11 items were included to assess the knowledge and use of approaches to aphasia assessment and rehabilitation among the participants. The survey results indicated that majority of participants had provided speech and language rehabilitation to patients with various types of aphasia and the percentage of participants who have provided rehabilitation is given in Table 1. The results indicated that 97.2% of participants reported that they provided treatment to Broca’s aphasia and on the lower side, 16.9% of participants reported that they provided treatment to patients with primary progressive aphasia.

In addition to the rehabilitation practices, few questions were also included about the diagnostic procedures used in aphasia diagnosis by the participants and results indicated 97.9% of participants reported use of validated diagnostic tests such as Western Aphasia Battery; 53.5% of participants also reported use of informal (translated/adapted versions of standard tests) in their clinical practice for diagnosis of aphasia. Further, 45.8% of participants reported use of formal screening tests for aphasia, while 29.6% of participants reported use of informal procedures for screening of aphasia among patients. The participants also described several challenges during the diagnosis of aphasia to an open-ended question, and the challenges were categorized under clinician-oriented, assessment methods, and client-oriented domains and the frequently reported challenges are described in Table 2.

Regarding the question on whether the participants select the treatment based on type of aphasia or aphasia as a single entity, 63.4% of participants reported that the treatment is planned based on type of aphasia, 3.5% of participants indicated that they consider aphasia as a single entity while planning treatments, and 33.1% of participants indicated that they consider both. Further, 9.2% of participants indicated that they use approach-based intervention to aphasia (such as functional approach, social approach, stimulation approach, etc.), whereas 27.5% of participants reported use of specific techniques (such as melodic intonation therapy, semantic feature analysis treatment, etc.) rather than approaches, and 63.4% reported use of both approach-based and technique-based interventions to aphasia rehabilitation. The responses to question on awareness about the six approaches to aphasia rehabilitation indicated that participant’s perceived awareness was highest for localization approach (91.5%) and lowest for cognitive neuropsychological approach (58.5%). The percentage of self-reported clinician awareness about each of the approaches is given in Table 3.

The participants were also asked to rate their knowledge level of each approach (very good, good, average, limited, very limited), confidence levels for each approach (very confident, confident, neutral, unconfident, very unconfident), and use of each approach (very frequently, frequently, occasionally, rarely, and very rarely) and the responses were coded on a five-point rating scale. The approaches included in the questions were stimulation approach, localization approach, neurolinguistic approach, cognitive neuropsychological approach, functional/pragmatic approach, and social approaches. The responses were statistically analyzed for mean, standard deviation and the results are given in Table 4.

**Figure 1:**
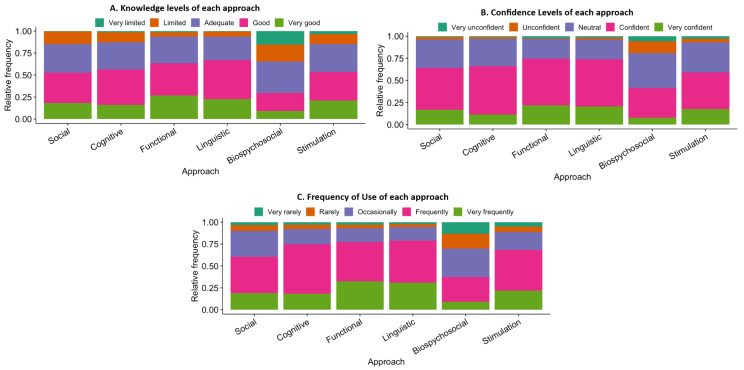
(**A**) Knowledge of SLPs for each approach. (**B**) Confidence levels of SLPs for each approach. (**C**) Frequency of Use of each approach.

The results of descriptive statistics of all approaches indicated mean score of 3.53 with standard deviation of 0.75 for knowledge; mean score of 3.70 and standard deviation of 0.63 for confidence; mean score of 3.71 and standard deviation of 0.72 for use of approaches. The pictorial representation of level of knowledge, confidence, and use are depicted in the Figure 1.

A multivariate GLM analysis was conducted to examine the influence of demographic variables of gender, workplace setting, academic qualifications, and experience on three self-reported measures of knowledge, confidence and use levels. The results of statistical analysis revealed statistically significant for knowledge (F = 3.00), confidence (F = 2.82), and use (F = 2.43) indicating that the model fits across all three constructs. Among the demographic and professional variables, gender showed significant differences with higher self-rated scores for males on knowledge (β = 0.365, *p* < 0.01), confidence (β = 0.283, *p* < 0.05), and use (β = 0.110, *p* < 0.05) compared to females. Although the academic qualifications, workplace settings, and geographical areas did show positive effects, the effects are statistically not significant. The model showed a moderate proportion of variance, with $R^{2}$ values of 0.27 for knowledge, 0.24 for confidence, and 0.20 for use. A correlation analysis was carried out using spearman correlation analysis between qualification, experience with knowledge, confidence and use of approaches and the results are given in Table 5.

The participants were also asked to rate the appropriateness of each approach for various phases of rehabilitation, such as the acute phase, inpatient rehabilitation, outpatient rehabilitation, and community care, and the results are given in Table 6.

### 3.2. Importance of Patient Education, Counselling, Follow-Up Process

The participants were asked if they would focus on educating or counselling the patient with aphasia and caregivers as part of rehabilitation, and results indicated that 99.3% of participants consider patient education and counselling for the patients and caregivers as very important. Further, 60.6% of participants reported that they would ask the patient with aphasia for follow-up after discharge, followed by 31% responding that they would recommend follow-up once in three months, and 7.7% of participants responded that they would recommend follow-up once in 6 months after discharge.

### 3.3. Bilingual and Multilingual

The survey included three questions related to bilingual and multilingual aspects of aphasia rehabilitation considering the linguistic diversity of India. For the question on treating bi- and multilingual patients with aphasia, 81.7% of the participants reported that they worked with bilingual and multilingual patients during their practice. Further, the participants were asked to provide basis of preference for choosing language among bi- and multilingual patients with aphasia. The results indicated that 70.4% of participants considered dominant language for treatment, followed by based on client preference with 57% (Table 7). Further, 59.2% of participants responded that they consider cross-linguistic treatment generalisation in aphasia rehabilitation.

### 3.4. Challenges to Aphasia Rehabilitation in Indian Context

The fifth section of survey included questions related to the challenges for clinicians and challenges for clients with aphasia for rehabilitation through open-ended questions. The responses were validated and the most perceived challenges by the participants are given in Table 8.

Further, 59.2% of respondents indicated that they have used various apps or softwares for aphasia rehabilitation and 56.3% of participants have used tele-rehabilitation methods for aphasia rehabilitation. The participants were also asked for future directions to aphasia rehabilitation in Indian context through an open-ended question and the responses of participants have been summarized in Table 9.

## 4. Discussion

The present research study aimed to investigate the existing practices, challenges, and future directions in aphasia rehabilitation by SLPs in Indian context. The study was carried out using a web-based questionnaire to gather responses from speech-language pathologists engaged in assessment and treatment of PWA across clinical, research, and academic settings in India. The questionnaire comprised 32 questions distributed across five domains: demographic and professional details; knowledge and use of approaches to aphasia rehabilitation; importance of patient education, counselling, and follow-up processes; bilingual and multilingual contexts; and challenges to aphasia rehabilitation within the Indian context.

### 4.1. Knowledge and Use of Approaches to Aphasia Rehabilitation

Formulating diagnosis is an essential step in planning rehabilitation for any communication disorder including aphasia. Hence, the present study included questions related to diagnostic practices by speech-language pathologists in Indian context given the nature of multilingual and multicultural nature of India. These results indicated significant challenges in diagnostic procedures used for assessment of aphasia in Indian context which warrants developing and validating more language specific formal assessment batteries for screening and diagnosis of aphasia in Indian context as also reported in previous studies [4,29,30,31]. Further, these challenges are not just limited to Indian context but are also extended to other nations with immigrants from culturally and linguistically backgrounds [34]. The second significant challenge for assessment and diagnosis of aphasia is related to the PWA in which many of the participants reported that presence of associated symptoms such as hemiplegia, psychological issues such as depression, poor mental status, lack of awareness, emotional instability, unsupportive caregivers as significant challenges in their practice. These challenges were also reported by studies from Turkey [35], United Kingdom [36], Switzerland [3], Sweden [37]. Participants also reported that language barriers, differential diagnosis, lack of time due to increased case load, and so on under clinician-related challenges in diagnostic process of aphasia.

The results also indicated a varied levels of awareness of various approaches which are widely used globally. This warrants more continuing educational and hands-on training programs for the speech language pathologists about the recent approaches in aphasia rehabilitation in Indian context frequently to enable to clients to be more efficient in using wide range of evidence-based approaches [38,39]. The participants were also asked to rate their knowledge, confidence and frequency of use of each approach on a 5-point scale and the results of self-reported ratings revealed highest level of knowledge for linguistic and functional approaches with least knowledge about biopsychosocial approach. On confidence levels, the participants reported highest confidence levels for functional approach and lowest confidence for biopsychosocial approach. Further, participants reported very frequent use of linguistic approach and used biopsychosocial approach very rarely in their practice.

This may indicate limited self-reported awareness of the biopsychosocial approach among speech language pathologists in Indian context although the approach has been widely advocated for goal setting for PWAs with strong evidence-based practice [40]. Similar results were also reported by Rose et al. [2] among Australian speech language pathologists with highest level of knowledge, confidence and use for functional approaches and lowest knowledge and confidence for therapy for culturally and linguistically diverse clients. Rose [40] elaborated on the importance of using biopsychosocial approach to patients with aphasia through evidence-based practices, utilizing technological advancements, incorporating multidisciplinary approaches to support psychological wellbeing of the patients and a comprehensive intervention to tackle multiple goals. Further, the correlation analysis revealed low positive correlation between experience with the knowledge, confidence and use of approaches, whereas significant correlation was observed between qualification and use of approaches only. These results indicate that the experienced speech language pathologists have reliable knowledge, confidence and use of approaches to aphasia rehabilitation rather than educational qualification as reported by participants. The results also revealed that participants prefer stimulation approach followed by functional approach in acute stage rehabilitation; functional and linguistic approaches in inpatient rehabilitation; cognitive neuropsychological approach followed by biopsychosocial approaches in outpatient rehabilitation; and social approach in community rehabilitation. These results are in contrast with the study in Australia by Rose et al. [2] where SLPs preferred augmentative and alternative communication (AAC) as most preferred method in acute stage of rehabilitation and in nursing homes; individual work and partner training as most preferred during inpatient rehabilitation; social and life participation approach in outpatient rehabilitation and community care.

### 4.2. Patient Education, Counselling and Follow-Up Process, Bilingual and Multilingual Practices

The results of the present study provided important insights into the current practices followed by SLPs in Indian context regarding importance of patient education, counselling, follow-up process and services to bilingual and multilingual patients. The participants of the study reported that they consider the patient education and counselling plays significant role in aphasia rehabilitation in Indian context and the results are in line with the previous studies and frameworks of life participation approach to aphasia which emphasize the role of patient and caregiver education along with psychosocial support through counselling [41]. Regular follow-up is an essential component in successful rehabilitation which will help the clinicians to monitor the maintenance and generalization of improvements achieved during treatment and is considered as a significant part of aphasia rehabilitation. However, significant challenges were reported by the participants in Indian context in setting-up regular follow-up practices due to wide range of factors such as high caseloads, financial or logistic barriers as also reported in other middle and low-income countries [42].

On bilingual and multilingual practices followed by Indian SLPs, the results indicated that more than 80% of participants reported that they are involved in treating bilingual and multilingual PWAs reflecting the nature of linguistic diversity of India. However, significant challenges were reported by the participants in terms of availability of validated assessment batteries, limited perceived knowledge regarding rehabilitation of bilingual patients, specifically in choosing the language for treatment planning which were also highlighted in western context [43]. The participants also reported a significant gap in knowledge on effects of cross-linguistic treatment effects due to lack of evidence-based research findings, specifically considering the structure of various Indian languages. This warrants the need for future research studies focusing of establishing evidence-based practices for bilingual and multilingual patients [44].

### 4.3. Challenges and Future Directions

The present study sheds light on several challenges faced by clinicians in aphasia rehabilitation and these challenges were for both clinician and client with aphasia. The responses to the open-ended question on challenges for clinician in aphasia rehabilitation in Indian context revealed deficiency of materials for treatment in Indian languages, variability in patient needs, addressing associated cognitive deficits, time constraints, lack of awareness of most recent updates in aphasia rehabilitation among many others which must be addressed in Indian context. The similar challenges were also reported by Tiwari & Krishnan [32], and Pauranik et al. [4], however, these issues are yet to be resolved in Indian context. The focus of academic education should be more on training the students of speech language pathology with more practical skills required for their clinical practice at both undergraduate and postgraduate levels. Additionally, the premiere institutions may start short-term clinical fellowship programs for SLPs in aphasiology which may enhance clinical skills.

On the other hand, significant challenges for clients as reported by participants included psychosocial disturbances, financial constraints (lack of funding or insurance), availability of rehabilitation services, lack of support from family members for various reasons, pressure for returning to work, are listed as major challenges for clients with aphasia in Indian context. These challenges require support from both governmental and non-governmental organizations along with teaching institutions to effectively deal with these challenges such as by establishing speech and language therapy services at semi-urban areas, primary healthcare centres at towns, and other healthcare facilities. Further, the participants also reported several challenges for clients with aphasia including financial issues or unavailability of insurance facilities for patients with aphasia for rehabilitation. These challenges may be addressed through collaboration between government bodies and insurance agencies to effectively address financial constraints among PWAs. Similar findings were also reported by previous studies conducted in low- and middle-income countries (LMICs) where the rehabilitation services are limited and continuous services are challenging [45,46].

Further, the speech language pathologists and the researchers in India should focus on developing more assessment and treatment tools tailored to multilingual population of India and make the resources available for other clinicians which will enhance the rehabilitation services to the patients with aphasia. In addition, the speech language pathologists may incorporate tele-rehabilitation practices and make use of available apps or softwares to improve the availability of services to clients with aphasia. Additional research is needed to develop language-specific softwares and mobile apps for patients in different Indian languages by collaborating with other technological disciplines. The results of the study also revealed that there is a limited awareness among public about aphasia and its implications which indirectly affects the aphasia rehabilitation practices in India. The speech language pathologists should work on establishing aphasia support groups in different parts of India which will in turn improve communication between patients, caregivers, and professionals.

## 5. Conclusions

The present study explored self-reported clinical practices, perceived challenges, and suggested future directions by Speech language pathologists for aphasia rehabilitation in Indian context. Although limited number of responses were obtained from SLPs, the findings of the study provided a preliminary insight into current practices to both assessment and rehabilitation of PWAs along with the challenges for aphasia rehabilitation from SLP perspectives. While the profession of Speech-language pathology is in existence from past 50 years in India, participants of the study have reported significant challenges in both assessment and rehabilitation of PWAs in India. The major challenges include limited availability of standardized assessment tools and intervention materials in Indian languages in addition to the logistic issues. These challenges emphasize the need for developing culturally and linguistically appropriate resources in Indian context to enhance the aphasia rehabilitation services in India. Future research studies are warranted with larger samples of both clinicians and PWAs from diverse geographical areas to understand the practices and challenges for rehabilitation of PWAs in Indian context.

## Figures and Tables

**Table 1:** Percentage of Clinicians Providing Rehabilitation Services for Different Types of Aphasia in India.

Type of Aphasia	Clinicians Providing Rehabilitation (%)
Global aphasia	85.2
Broca’s aphasia	97.2
Transcortical motor aphasia	40.8
Mixed transcortical aphasia	19.0
Wernicke’s aphasia	76.1
Transcortical sensory aphasia	31.7
Conduction aphasia	36.6
Anomic aphasia	75.4
Primary progressive aphasia	16.9
Subcortical aphasia	21.8

**Table 2:** Challenges for assessment of aphasia in Indian context as reported by the participants.

Clinician-Oriented Challenges	Challenges Related to Assessment Methods	Client-Oriented Challenges
Ruling out cognitive communication deficits in patients with aphasia	Lack of availability of culturally and linguistically specific test and treatment protocols	Sometimes parents or guardians try to hide information regarding case history
Differential diagnosis, specifically at the initial stages	Limited availability of brief tests for aphasia besides bedside WAB	Patient cooperation issues
Time constraints in conducting formal tests and follow-up in private cases	Unavailability of test materials in regional languages	Difficulty in assessment especially with limitations of understanding
Difficulty in correlating subcortical lesions with aphasia types	Language-specific test tools; Time constraints in multispecialty hospitals; Referral delays	Patient non-cooperative and irritable attitude
Language barriers	Adapting questionnaire items for client’s understanding	Patients have additional physical impairments/trach patients or with weakness; unsupportive caregivers and family members; lack of motivation
	WAB is too lengthy; Needs shorter tests	Poor mental status, emotional instability
	Bilingual and multilingual patients with aphasia	While using formal test materials, questions may be too complex for patients from rural areas or who are illiterate
	Availability of test materials in dialects (Konkani, Tulu, Baery languages)	Patients with aphasia often experience frustration, depression, or anxiety affecting assessment responses
	Language used in formal diagnostic tests (WAB) is outdated; Sometimes results based on scores and observed symptoms mismatch	Challenges from caregivers
	Assessment is lengthy and takes multiple sessions for in-depth evaluation	Patient and caregiver behaviors
	Challenge in differential diagnosis	Literacy of patients while assessing reading and writing
		Sometimes lack of interest to answer; Emotional factors

**Table 3:** Clinician’s self-reported awareness of various approaches with description in aphasia rehabilitation.

Approach	Description of Approach	Awareness (%)
Stimulation approach	Focus on rehabilitation of PWA using repeated and structured auditory & multimodal language input to facilitate language recovery.	73.2
Localization approach	Focus on targeting deficits associated with specific brain lesions of language.	91.5
Neurolinguistic approach	Focus on targeting the affected linguistic components such as semantics, syntax, etc.	62.0
Cognitive neuropsychological approach	Focus on treating the underlying cognitive-linguistic processing deficits.	58.5
Functional/pragmatic approach	Focus on improving functional communication and conversational effectiveness.	82.4
Social approach	Focus on improving communication within social environments with emphasis on social inclusion and reducing the barriers affecting social interactions.	62.7

**Table 4:** Clinician’s perceived knowledge, confidence, and use of each approach by clinicians.

Approach	Knowledge			Confidence			Use		
	Mean	SD	Range	Mean	SD	Range	Mean	SD	Range
Stimulation approach	3.56	1.05	1–5	3.66	0.91	1–5	3.73	1.02	1–5
Linguistic approach	3.82	0.86	1–5	3.89	0.79	1–5	4.02	0.89	1–5
Biopsychosocial approach	2.88	1.17	1–5	3.24	0.97	1–5	3.02	1.16	1–5
Cognitive neuropsychological approach	3.58	0.93	1–5	3.73	0.71	1–5	3.82	0.88	1–5
Functional/pragmatic approach	3.82	0.93	1–5	3.91	0.81	1–5	4.00	0.95	1–5
Social approach	3.55	0.97	1–5	3.76	0.78	1–5	3.66	0.97	1–5

**Table 5:** Correlation between qualifications, experience of clinicians with their knowledge, confidence and use of various approaches.

	Knowledge	Confidence	Use
Qualification	0.100	0.106	0.179 **
Experience	0.233 **	0.154 **	0.206 *
Knowledge	–	0.682 *	0.638 **
Confidence	0.682 *	–	0.660 **
Use	0.638 *	0.660 **	–

* p<0.05; ** p<0.01.

**Table 6:** Clinician’s preferences for use of each approach at various stages.

Approach	Acute	Inpatient	Outpatient	Community
	n (%)	n (%)	n (%)	n (%)
Stimulation approach	40 (28.2)	58 (40.8)	37 (26.1)	7 (4.9)
Linguistic approach	26 (18.3)	58 (40.9)	49 (34.5)	9 (6.3)
Biopsychosocial approach	27 (19.0)	41 (28.9)	50 (35.2)	24 (16.9)
Cognitive neuropsychological approach	20 (14.1)	64 (45.0)	51 (36.0)	7 (4.9)
Functional/pragmatic approach	31 (21.9)	58 (40.9)	47 (33.0)	6 (4.2)
Social approach	21 (14.8)	22 (15.5)	44 (31.0)	55 (38.7)

**Table 7:** Percentage of clinician’s preferences for language selection for bilingual and multilingual patients.

Language Selection Criteria	Percentage (%)
Native language	49.3
Dominant language	70.4
Weaker language	6.3
All languages known	12.0
Based on the client’s preference	57.0
Based on the type and severity of language impairment	27.5

**Table 8:** Challenges to aphasia rehabilitation in Indian context as reported by the participants.

Challenges for Clinicians in Aphasia Rehabilitation	Challenges for Patients with Aphasia for Rehabilitation
Patient Expectations are quite high with shorter span of period	chronic nature of aphasia, follow-up practices; and financial issues to continue treatment
Finding stimuli as client reaches syntactic. Reading and writing tasks	Adaptation to the society and life participation. Returning to work
The most significant challenges in aphasia rehabilitation include individual variability in patient needs, maintaining motivation and engagement, and addressing cognitive/emotional co-morbidities. Involving caregivers effectively while managing their expectations is also crucial. Limited resources and time constraints further complicate therapy	significant challenges such as frustration and emotional distress from communication difficulties, difficulty relearning language skills, and social isolation due to reduced ability to interact with others. They may also struggle with adjusting to the long-term nature of recovery and dealing with cognitive impairments that accompany aphasia.
lack of resources (time, space, materials) as a major challenge to effective service provision.	Logistics; Lack of funding and insurance; Non-available quality therapists; Lack of home-based training
Follow up and proper practice of the given approaches by the family/care taker.	Communication, Social acceptance including in the family, Emotional frustration and Stress to recover quickly and resume work.
Poor generalization	Family members support, lack of motivation
Motivating the patient for therapy	Lack of understanding of the problem therefore lack of patience
The lack of awareness of most recent updates in aphasia rehab	Emotional distress, communicational barrier, demotivating surrounding
Convincing that recovery is slow and they have to keep patience; Treatment approaches	Lack of professional or ASLP near to their location
1. Translating researcher into clinical practices; 2. Maintaining long term treatment adherence; 3. Managing emotional challenges of the patients	Consistency in taking therapy, socioeconomic inability and lack of multidisciplinary approach
Sometimes I get Stuck at a point where I don’t know what next?	
Patients not being regular with practice and with low motivation	
Convincing families especially client regarding the steps involved in therapy.	
I have not had much experience and exposure to various treatment approaches. Hence, I feel I am very much fixed/stuck with a few approach directions for the patients.	
Finding support groups for individuals and their families to understand different aspects of Aphasia and Caregiver support for regular therapy	
Poor referral during acute phases, poor availability of services, lack of insurance for SLP services, lack of rehabilitation centres	

**Table 9:** Future directions in aphasia rehabilitation as self-reported by participants.

Future directions in aphasia rehabilitation in India include developing multilingual, culturally relevant therapy tools, expanding teletherapy for wider access, and integrating technology such as AI for personalized interventions. Research will focus on creating cost-effective, community-based rehabilitation models and improving awareness and training for healthcare providers.	Improving the tele rehabilitation service for individuals at remote areas or conducting camps and assessing and providing timely intervention for PWA at community level. Including more of social approaches in communication.
Future in-depth studies on these issues incorporating SLPs as well as PWA and their close relatives may provide us more insight into these issues. The outcomes of such studies may serve as the backbones of the policy-making by the concerned authorities towards the rehabilitation of people with aphasia in India	Apps like Constant Therapy, if validated in Indian Languages can help in better generalisation of language skills at home. Technologies like Transcranial Direct Current Stimulation (TDCS), which has been shown to provide better language treatment outcomes in countries like U.S can emerge as a regular SLP practise in India (Research on its efficacy is already going on in some of the academic institutions in India)
Apps for rehabilitation in Indian language	Need extensive evidence-based research on bilingual aphasia treatments.
Affordable and Accessible AAC, Tele Rehab and digital Platform, Integrating Holistic & Family centred.	Raising awareness about aphasia and its impact on communication with health care professionals, caregivers, and public.
Quality of life-based treatment approaches	Hands-on program/seminars is required.
I think aphasia research has reached a plateau. Only neuromodulation or naming is only discussed in most of research. Severe aphasias are never discussed.	Rehabilitation with AI and technology incorporated softwares and devices.
1. Development and validation of culturally and linguistically specific tools; 2. Inclusive rehabilitation using technology; 3. Qualitative research; 4. Well controlled RCTs	Awareness among private settings for immediate speech therapy services; Evidence of tools used; Therapy demonstrations
Maybe a lot of workshops/awareness about approaches and techniques in needed for SLPs. As far as research is concerned, I believe the diagnostic part of it needs to be focused more.	It needed to be upgraded more towards the overall approach and improvement in overall quality of life rather than only on speech and language
In Indian context, research must be done to develop Indian assessment tools, bilingual assessment tools. A treatment manual can be developed which would serve as a guide to the SLPs	Evidence Based Practice, App based intervention, Efficacy studies, Implementation of WHO ICF concept of framework in Rehabilitation
Encouraging collaboration among speech-language pathologists, neurologists, psychologists, and other professionals to create holistic treatment. Enhancing training programs for healthcare professionals to ensure they are well-versed in current evidence-based practices for aphasia treatment.	Developing rehabilitation programs that incorporate local languages, dialects, and cultural nuances to make therapy more effective and relatable for patients. Use of teletherapy and digital tools to reach patients in remote areas, ensuring broader access to rehabilitation services.

## Data Availability

Data will be available at first author, upon request data can be obtained.

## References

[1] Fama M., Turkeltaub P. (2014). Treatment of Poststroke Aphasia: Current Practice and New Directions. Semin. Neurol..

[2] Rose M., Ferguson A., Power E., Togher L., Worrall L. (2014). Aphasia Rehabilitation in Australia: Current Practices, Challenges and Future Directions. Int. J. Speech-Lang. Pathol..

[3] Engelter S.T., Gostynski M., Papa S., Frei M., Born C., Ajdacic-Gross V., Gutzwiller F., Lyrer P.A. (2006). Epidemiology of Aphasia Attributable to First Ischemic Stroke: Incidence, Severity, Fluency, Etiology, and Thrombolysis. Stroke.

[4] Pauranik A., George A., Sahu A., Nehra A., Paplikar A., Bhat C., Krishnan G., Kaur H., Saini J., Suresh P. (2019). Expert Group Meeting on Aphasia: A Report. Ann. Indian Acad. Neurol..

[5] Lazar R.M., Minzer B., Antoniello D., Festa J.R., Krakauer J.W., Marshall R.S. (2010). Improvement in Aphasia Scores After Stroke Is Well Predicted by Initial Severity. Stroke.

[6] Laska A.C., Hellblom A., Murray V., Kahan T., Von Arbin M. (2001). Aphasia in Acute Stroke and Relation to Outcome. J. Intern. Med..

[7] Wilson S.M., Eriksson D.K., Brandt T.H., Schneck S.M., Lucanie J.M., Burchfield A.S., Charney S., Quillen I.A., De Riesthal M., Kirshner H.S. (2019). Patterns of Recovery From Aphasia in the First 2 Weeks After Stroke. J. Speech Lang. Hear. Res..

[8] Hanlon R.E., Lux W.E., Dromerick A.W. (1999). Global Aphasia without Hemiparesis: Language Profiles and Lesion Distribution. J. Neurol. Neurosurg. Psychiatry.

[9] Kristinsson S., Den Ouden D.B., Rorden C., Newman-Norlund R., Neils-Strunjas J., Fridriksson J. (2022). Predictors of Therapy Response in Chronic Aphasia: Building a Foundation for Personalized Aphasia Therapy. J. Stroke.

[10] Hope T.M.H., Seghier M.L., Leff A.P., Price C.J. (2013). Predicting Outcome and Recovery after Stroke with Lesions Extracted from MRI Images. NeuroImage Clin..

[11] Busby N., Newman-Norlund S., Sayers S., Rorden C., Newman-Norlund R., Wilmskoetter J., Roth R., Wilson S., Schwen-Blackett D., Kristinsson S. (2024). Regional Brain Aging: Premature Aging of the Domain General System Predicts Aphasia Severity. Commun. Biol..

[12] Cramer S.C. (2008). Repairing the Human Brain after Stroke: I. Mechanisms of Spontaneous Recovery. Ann. Neurol..

[13] El-Tallawy H.N., Gad A.H.E.S., Ali A.M., Abd-El-Hakim M.N. (2019). Relative Frequency and Prognosis of Vascular Aphasia (Follow-up at 3 Months) in the Neurology Department of Assiut University Hospital. Egypt. J. Neurol. Psychiatry Neurosurg..

[14] Mc Menamin R., Tierney E., Mac Farlane A. (2015). Addressing the Long-Term Impacts of Aphasia: How Far Does the Conversation Partner Programme Go?. Aphasiology.

[15] Meinzer M., Djundja D., Barthel G., Elbert T., Rockstroh B. (2005). Long-Term Stability of Improved Language Functions in Chronic Aphasia After Constraint-Induced Aphasia Therapy. Stroke.

[16] Holland A., Fromm D., Forbes M., MacWhinney B. (2017). Long-Term Recovery in Stroke Accompanied by Aphasia: A Reconsideration. Aphasiology.

[17] Harvey D.Y., Parchure S., Hamilton R.H. (2022). Factors Predicting Long-Term Recovery from Post-Stroke Aphasia. Aphasiology.

[18] Brady M.C., Godwin J., Enderby P., Kelly H., Campbell P. (2016). Speech and Language Therapy for Aphasia After Stroke: An Updated Systematic Review and Meta-Analyses. Stroke.

[19] Bhogal S.K., Teasell R., Speechley M. (2003). Intensity of Aphasia Therapy, Impact on Recovery. Stroke.

[20] Zhang J., Yu J., Bao Y., Xie Q., Xu Y., Zhang J., Wang P. (2017). Constraint-Induced Aphasia Therapy in Post-Stroke Aphasia Rehabilitation: A Systematic Review and Meta-Analysis of Randomized Controlled Trials. PLoS ONE.

[21] Shewan C.M., Kertesz A. (1984). Effects of Speech and Language Treatment on Recovery from Aphasia. Brain Lang..

[22] Bowen A., Hesketh A., Patchick E., Young A., Davies L., Vail A., Long A.F., Watkins C., Wilkinson M., Pearl G. (2012). Effectiveness of Enhanced Communication Therapy in the First Four Months after Stroke for Aphasia and Dysarthria: A Randomised Controlled Trial. BMJ.

[23] Nouwens F., De Lau L.M., Visch-Brink E.G., Van De Sandt-Koenderman W.M.E., Lingsma H.F., Goosen S., Blom D.M., Koudstaal P.J., Dippel D.W. (2017). Efficacy of Early Cognitive-Linguistic Treatment for Aphasia Due to Stroke: A Randomised Controlled Trial (Rotterdam Aphasia Therapy Study-3). Eur. Stroke J..

[24] Breitenstein C., Grewe T., Flöel A., Ziegler W., Springer L., Martus P., Huber W., Willmes K., Ringelstein E.B., Haeusler K.G. (2017). Intensive Speech and Language Therapy in Patients with Chronic Aphasia after Stroke: A Randomised, Open-Label, Blinded-Endpoint, Controlled Trial in a Health-Care Setting. Lancet.

[25] Larweh G., Owusu Antwi A.A., Owusu E.A., Tagoe T.A. (2025). Exploring Benefits of Speech and Language Therapy Interventions for Post-Stroke Aphasia Rehabilitation: A Qualitative Study. Heliyon.

[26] Katz R.C., Hallowell B., Code C., Armstrong E., Roberts P., Pound C., Katz L. (2000). A Multinational Comparison of Aphasia Management Practices. Int. J. Lang. Commun. Disord..

[27] Chazhikat E., Olness G.S., Code C. Awareness of Aphasia and Aphasia Services in South India. Proceedings of the 2012 Annual Convention of the American Speech-Language-Hearing Association.

[28] Kertesz A. (2006). Western Aphasia Battery—Revised.

[29] Chandana S.S.M., Goswami S.P. (2025). Survey on Current Practices in the Clinical Assessment of Persons with Aphasia in India. Speech Lang. Hear..

[30] Kiran S., Cherney L.R., Kagan A., Haley K.L., Antonucci S.M., Schwartz M., Holland A.L., Simmons-Mackie N. (2018). Aphasia Assessments: A Survey of Clinical and Research Settings. Aphasiology.

[31] Paplikar A., Iyer G., Varghese F., Alladi S., Pauranik A., Mekala S., Kaul S., Sharma M., Dhaliwal R., Saroja A. (2020). A Screening Tool to Detect Stroke Aphasia: Adaptation of Frenchay Aphasia Screening Test (FAST) to the Indian Context. Ann. Indian Acad. Neurol..

[32] Tiwari S., Krishnan G. (2011). Aphasia rehabilitation in India: A preliminary survey of Speech-Language pathologists. J. All India Inst. Speech Hear..

[33] Oducado R.M. (2020). Survey instrument validation rating scale. SSRN J..

[34] Gibson D., Braun P., Benham C., Mason F. (2021). Projections of Older Immigrants: People from Culturally and Linguistically Diverse Backgrounds, 1996–2026, Australia.

[35] Maviş I. (2007). Perspectives on Public Awareness of Stroke and Aphasia among Turkish Patients in a Neurology Unit. Clin. Linguist. Phon..

[36] Flynn L., Cumberland A., Marshall J. (2009). Public Knowledge about Aphasia: A Survey with Comparative Data. Aphasiology.

[37] Johansson M.B., Carlsson M., Sonnander K. (2011). Working with Families of Persons with Aphasia: A Survey of Swedish Speech and Language Pathologists. Disabil. Rehabil..

[38] Page C., Howell D. (2015). Current Clinical Practice of Speech-Language Pathologists Who Treat Individuals with Aphasia. J. Interact. Res. Commun. Disord..

[39] Monnelly K., Marshall J., Dipper L., Cruice M. (2023). Intensive and Comprehensive Aphasia Therapy—A Survey of the Definitions, Practices and Views of Speech and Language Therapists in the United Kingdom. Int. J. Lang. Commun. Disord..

[40] Rose M.L. (2023). Elizabeth Usher Memorial Lecture: Beyond Our Practice Borders—Using a Biopsychosocial Framework to Improve Long-Term Outcomes for People Living with Aphasia. Int. J. Speech-Lang. Pathol..

[41] Chapey R., Duchan J.F., Elman R.J., Garcia L.J., Kagan A., Lyon J.G., Simmons Mackie N. (2000). Life participation approach to aphasia: A statement of values for the future. ASHA Lead..

[42] Kiran S., Thompson C.K. (2019). Neuroplasticity of language networks in aphasia: Advances, updates, and future challenges. Front. Neurol..

[43] PArAdis M. (2001). The need for awareness of aphasia symptoms in different languages. J. Neurolinguist..

[44] Ansaldo A.I., Marcotte K., Scherer L., Raboyeau G. (2008). Language therapy and bilingual aphasia: Clinical implications of psycholinguistic and neuroimaging research. J. Neurolinguist..

[45] Bright T., Kuper H. (2018). A systematic review of access to general healthcare services for people with disabilities in low and middle income countries. Int. J. Environ. Res. Public Health.

[46] Cieza A., Causey K., Kamenov K., Hanson S.W., Chatterji S., Vos T. (2020). Global estimates of the need for rehabilitation based on the Global Burden of Disease study 2019: A systematic analysis for the Global Burden of Disease Study 2019. Lancet.

